# Whole genome sequencing reveals extended natural transformation in *Campylobacter* impacting diagnostics and the pathogens adaptive potential

**DOI:** 10.1038/s41598-020-60320-y

**Published:** 2020-02-28

**Authors:** Julia C. Golz, Lennard Epping, Marie-Theres Knüver, Maria Borowiak, Felix Hartkopf, Carlus Deneke, Burkhard Malorny, Torsten Semmler, Kerstin Stingl

**Affiliations:** 1 German Federal Institute for Risk Assessment, Department of Biological Safety, National Reference Laboratory for Campylobacter, Berlin, Germany; 2 German Federal Institute for Risk Assessment, Department of Biological Safety, Study Centre for Genome Sequencing and Analysis, Berlin, Germany; 3 Robert Koch Institute, Microbial Genomics, Berlin, Germany

**Keywords:** Bacterial genomics, Food microbiology, Pathogens

## Abstract

*Campylobacter* is the major bacterial agent of human gastroenteritis worldwide and represents a crucial global public health burden. Species differentiation of *C. jejuni* and *C. coli* and phylogenetic analysis is challenged by inter-species horizontal gene transfer. Routine real-time PCR on more than 4000 *C. jejuni* and *C. coli* field strains identified isolates with ambiguous PCR results for species differentiation, in particular, from the isolation source eggs. K-mer analysis of whole genome sequencing data indicated the presence of *C. coli* hybrid strains with huge amounts of *C. jejuni* introgression. Recombination events were distributed over the whole chromosome. MLST typing was impaired, since *C. jejuni* sequences were also found in six of the seven housekeeping genes. cgMLST suggested that the strains were phylogenetically unrelated. Intriguingly, the strains shared a stress response set of *C. jejuni* variant genes, with proposed roles in oxidative, osmotic and general stress defence, chromosome maintenance and repair, membrane transport, cell wall and capsular biosynthesis and chemotaxis. The results have practical impact on routine typing and on the understanding of the functional adaption to harsh environments, enabling successful spreading and persistence of *Campylobacter*.

## Introduction

Since 2005, *Campylobacter* is the major zoonotic agent in the European Union, causing 250,161 confirmed campylobacteriosis cases in 2017^1^. Around one third of the cases can be directly attributed to handling, preparation and consumption of broiler meat^2^. Measures for *Campylobacter* reduction focus on virulence mechanisms and persistence factors, enabling the pathogen to successfully circulate within the food chain.

Typing of *Campylobacter* by species differentiation methods and by multi-locus sequence typing (MLST) has become key tools for diagnostics and source attribution. Specific gene targets have proven stable and were, therefore, chosen for this purpose. Two of commonly used species differentiation markers^3–5^ are *mapA*, a fitness factor in chicken colonization^6^ and *ceuE* playing a role in iron acquisition^7^. For MLST, central enzymatic functions, which are conserved in the genome were defined^8^ and are commonly used for phylogenetic analysis.

It was shown that high level of interspecies transfer of genetic material can occur between *C. jejuni* and *C. coli*^9^. Adaptation to hosts can modulate the gene pool and allele variants and was suggested to be of more relevance than geographical location^10^.

Here we identified extensive interspecies gene transfer from *C. jejuni* to *C. coli*, impairing species differentiation and MLST analysis. Whole genome sequencing revealed that these hybrid strains shared *C. jejuni* gene variants, involved in stress response. Since the hybrids had predominantly been isolated from egg shells, we suggest that gene variations due to *C. jejuni* sequence introgression might have been a consequence of selection of survivors in a harsh environment.

## Results

Isolates had been collected from food and animal matrices during routine sampling or zoonosis monitoring by the Federal State Laboratories between January 2016 and December 2018 according to ISO 10272^11^. The isolates were analysed by real-time PCR in the German National Reference Laboratory for *Campylobacter*^4,5^. The target for *C. jejuni* is a fragment of *mapA*, coding for an outer membrane protein. The *C. coli* specific target *ceuE* encodes the enterochelin uptake substrate-binding protein, involved in iron acquisition^3^. Out of 4.335 *C. jejuni* and *C. coli* isolates, 31 delivered ambiguous PCR results (0.72%). Ambiguous PCR results were defined by either amplification of both specific targets for *C. jejuni* and *C. coli* with similar Ct values (Cj/Cc mix, 28/31) or by no amplification at all (none, 3/31). A subsequent gel-based multiplex PCR^12^, targeting the *hipO* gene of *C. jejuni* and the *glyA* gene of *C. coli*, indicated that all of these isolates belonged to the species *C. coli*.

The 31 strains with ambiguous real-time PCR signal had been isolated from poultry meat, turkey cecum or skin and eggs. The Federal State Laboratories either did not report any species (9/31), correctly identified *C. coli* (20/31) or in one case falsely reported *C. jejuni* (1/31). Surprisingly, when we compared the number of strains with ambiguous real-time PCR result with the total number of isolates analysed during the same time of collection, proportionally the highest percentage of strains with ambiguous qPCR results was derived from eggs (Table 1), although the total number of analysed eggs was low.

**Table 1:** Distribution of *Campylobacter* isolation sources of isolates with ambiguous PCR results.

Isolation source*	# total isolates investigated (*Cj* and *Cc*)	# isolates with ambiguous PCR	% of isolates with ambiguous PCR relative to *Cj* and *Cc*	# total *Cc* isolates	% of isolates with ambiguous PCR relative to *Cc*
eggs	39	5	12.8	11	45.5
duck meat	63	1	1.6	20	5
chicken meat	1245	7	0.6	281	2.5
turkey meat	351	5	1.4	95	5.3
turkey cecum/skin	777	13	1.7	414	3.1

*Cj, C. jejuni; Cc, C. coli;* *isolates were obtained in the years 2016–2018; isolation source are displayed, from which isolates with ambiguous PCR results were obtained. Further 1860 isolates from other sources did not result in an ambiguous PCR result and were omitted from the table.

We characterized these isolates by whole genome sequence analysis. In addition, further *C. coli* isolates from previous years (n = 26, 2009–2015) were included. As the prevalence of strains with ambiguous qPCR was highest from eggs, we included those from eggs and additional isolates from laying hens, chicken meat and pig feces.

**Figure 1:**
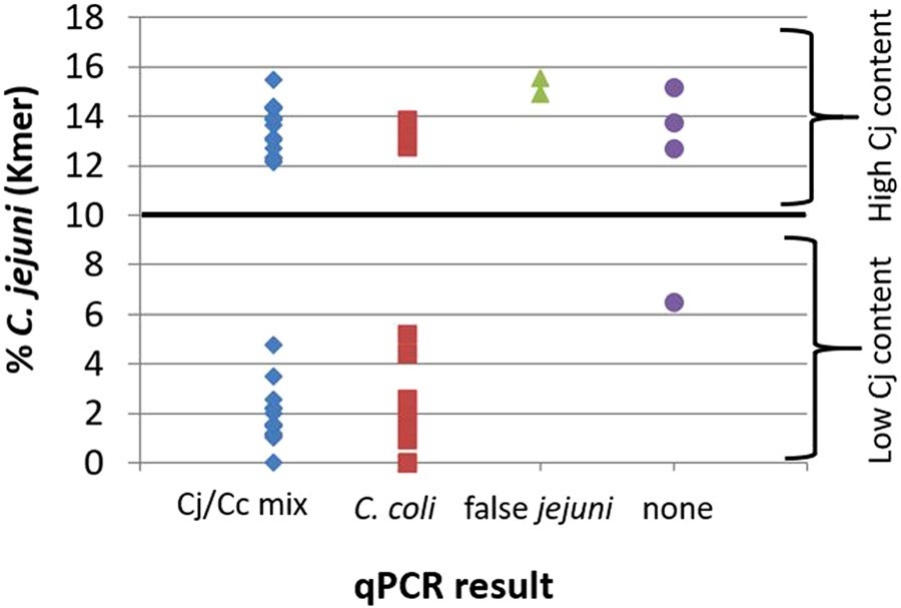
Percentage of *C. jejuni* genome content in *C. coli* isolates detected by k-mer analysis, categorized by qPCR result using *mapA*/*ceuE* as targets. Cj/Cc mix, both targets for *C. jejuni* and *C. coli* were amplified; *C.* *coli*, *C. coli* was correctly detected; false *jejuni*, *C. coli* was falsely detected as *C. jejuni*.; none, none of the targets was amplified.

**Figure 2:**
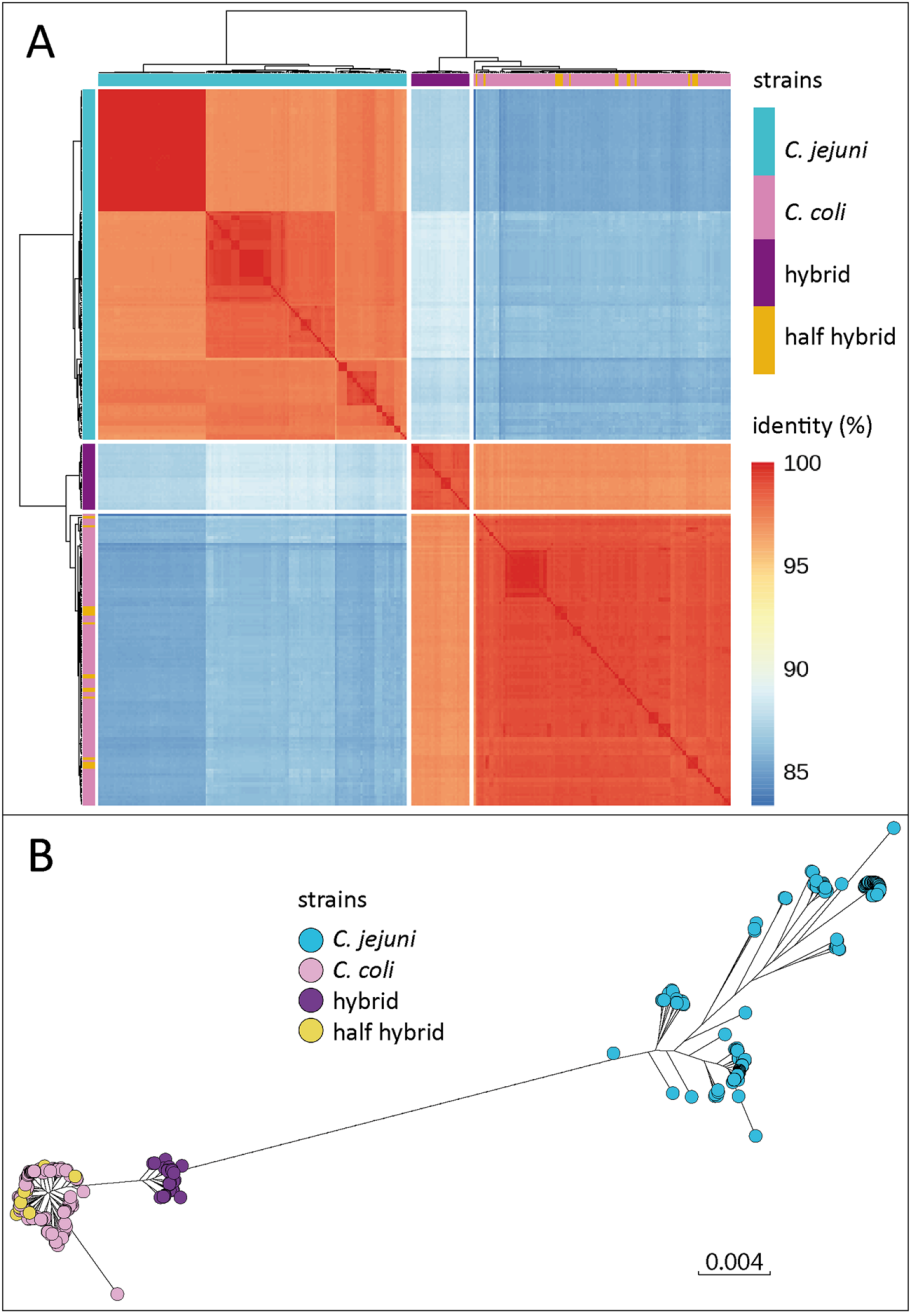
Relatedness of *C. jejuni* (turquoise), *C. coli* (pink), hybrid strains (purple) and half hybrid strains (mustard) according to ANI (**A**) and core genome analysis using Roary (**B**). (**A**) heatmap visualization of ANI values across all isolates. Hybrid strains form a separate cluster but still share ~97% ANI with *C. coli*. Half hybrid isolates are spread across the *C. coli* population. (**B**) phylogeny of the *Campylobacter* core genomes based on Roary analysis. The branch length between *C. coli*, including hybrid and half hybrid strains and *C. jejuni* has been shortened for better visualization.

We performed a k-mer based analysis using the KmerFinder 3.1 (CGE, DTU, Denmark)^13–15^. For a typical *C. coli* it is expected that the k-mers match to different *C. coli* reference genomes. However, as expected from the real-time PCR results, the k-mers of the input *C. coli* sequences with ambiguous PCR results also exhibited *C. jejuni* genomic content. Different percentages of k-mers matching to *C. jejuni* reference genomes were observed, ranging from 0 (undetectable) to 15.5% (Fig. 1). Also correctly PCR-identified *C. coli* exhibited various amounts of *C. jejuni* content (Fig. 1, red squares). From the latter, those with the highest *C. jejuni* content (>10%) were exclusively from eggs, two others with *C. jejuni* content between 4.4–5.3% were from chicken meat and turkey cecum. These isolates apparently harboured an extended amount of *C. jejuni* sequences but maintained *C. coli* sequences at *mapA* and *ceuE*. In total, 29 *C. coli* isolates with a k-mer percentage of more than 10% *C. jejuni* content (Fig. 1, categorized *C. coli* with high *C. jejuni* content, named “hybrids”) were identified. “Half hybrid” strains were defined as harbouring <10% *C. jejuni* sequences but displaying an ambiguous qPCR result. According to MALDI-TOF analysis, all hybrid and half hybrid strains belonged to *C. coli* with a score of ≥ 2.000, which was previously validated as indicative for correct species identification of *Campylobacter* spp.^16–18^. In order to clarify genetic relationship of “hybrid” and “half hybrid” strains within the *Campylobacter* population we performed an average nucleotide identity (ANI) analysis using the tool FastANI^19^. Population and ANI studies of recent years showed that organisms sharing at least 95% ANI among themselves are defined to be of the same species^20,21^. Results of the ANI analysis were visualized in Fig. 2A and reveal that “hybrid“ strains form a separate cluster but still share an ANI of 96.95% with *C. coli* isolates, proving them to be part of the *C. coli* population. In comparison, the ANI in the *C. jejuni* population ranges from 97% to 100% and shares 87.92% ANI with the “hybrid” isolates. “Half hybrid” strains are closely related with *C. coli* (98.96% ANI) and cannot be separated by ANI. A very similar observation can be made while looking at the core genome phylogeny in Fig. 2B based on Roary analysis using a sequence identity of at least 80%. The “half hybrid” isolates are found among the original *C. coli* population, whereas the hybrid strains form a separated, but still closely related group nearby *C. coli*. In general, both analyses show that the diversity/identity between *C. coli* including “hybrids” is similar than the diversity within the *C. jejuni* population, confirming that the hybrid strains indeed belong to the species *C. coli*.

**Figure 3:**
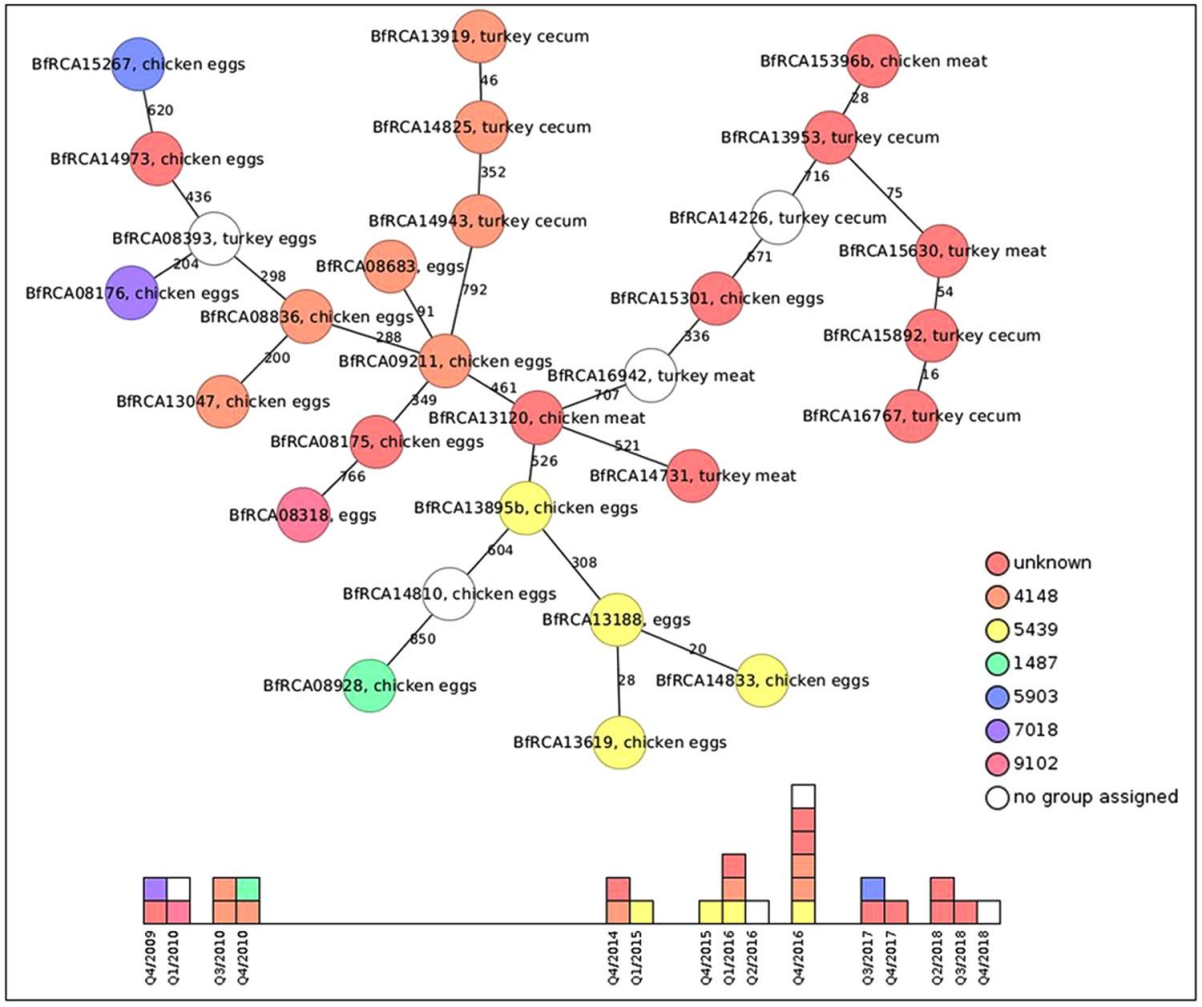
The 29 *C. coli* with at least 10% *C. jejuni* genome content are not related and isolated from different locations and at distinct timepoints. Minimum spanning tree of cgMLST analysis, based on 1343 core genes defined previously^22^. Circles represent *C. coli* isolates; numbers illustrate allele differences between nearest neighbours; isolation matrix is indicated as text; colour code for MLST ST-type as depicted in the legend. Unknown, new ST-type; no group assigned, at least one allele of the 7 housekeeping genes is new. New alleles and ST-types were submitted to PuMLST and numbers are shown in Supplementary Table S1.

We further asked whether the hybrid strains were of clonal origin and disseminated upon the/multiple horizontal *C. jejuni* gene transfer events had occurred. For this purpose, the MLST type was analysed based on the seven housekeeping genes (^8^, PubMLST.org). As visualized in Fig. 3 the isolates belonged to different sequence types, from which only two could be attributed to any known clonal complex (both ST-1150 complex), implicating that none of the isolates belonged to the CC-828 complex, which is the most abundant clonal complex of *C. coli*. More specific sequence analysis showed that these housekeeping genes were also affected by *C. jejuni* sequence introgression (see below). Thus, MLST typing apparently has its limitations in those *Campylobacter* with substantial horizontal gene transfer activity.

Analysis via Ridom Seqsphere+ software using allele-based cgMLST^22^ of 1343 genes indicated that the 29 *C. coli* hybrids were in majority unrelated. The median number of allele differences between the nearest neighbour was 343 (¼ of all analysed genes, Fig. 3). Thus, most of the strains displayed a phylogenetically diverse origin. This was substantiated by different isolation dates, ranging from 2009 to 2018 and different isolation locations from six federal states of Germany. Taken together, these data indicate that horizontal gene transfer from *C. jejuni* independently occurred in the *C. coli* hybrids.

### Where did *C. jejuni* introgression take place

**Figure 4:**
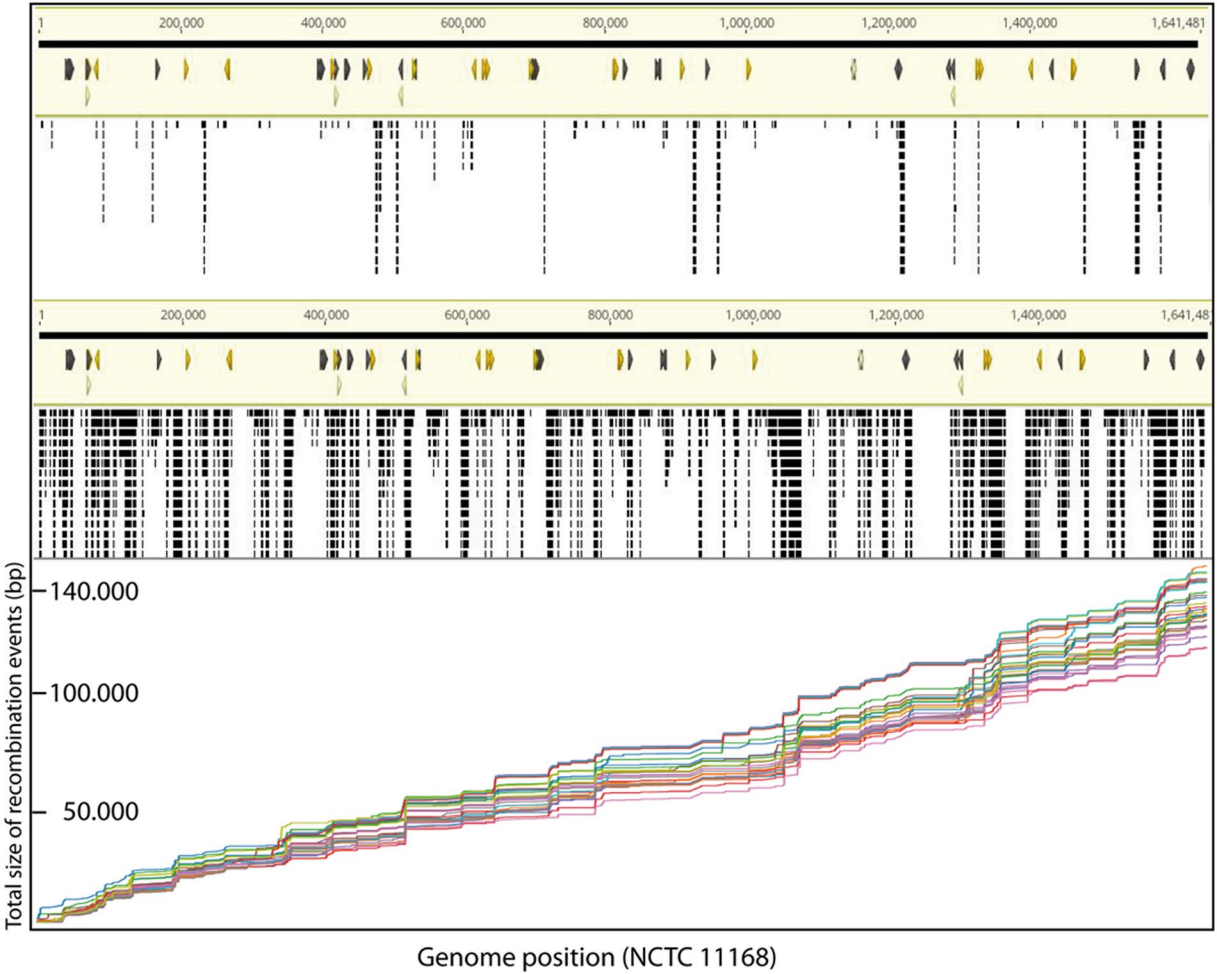
Cc/Cj hybrid strains show recombination events of *C. jejuni* sequences throughout the chromosome. k-mer mapping of *C. jejuni* sequences from two example strains to reference sequence of *C. jejuni* NCTC 11168 (in light yellow, visualized linearly from left (1^st^ base) to right (1,64 Mb)), visualized with Geneious Prime 2019.2.1. Upper panel, “half hybrid” BfR-CA-15281 (<10% *C. jejuni* introgression); middle panel, “hybrid” BfR-CA-14731 (>10% *C. jejuni* introgression); lower panel, cumulative plot of recombination events in 29 “hybrid” strains indicate common locations of *C. jejuni* sequence introgression. Black bars, k-mer matching at the indicated location in the *C. jejuni* genome.

An in-house k-mer analysis was performed. The *C. coli* sequences with >10% of *C. jejuni* introgression were split into 16-mers or 31-mers, which were compared against a set of 95 complete *C. coli* genomes obtained from the NCBI database and 18 further *C. coli* strains without PCR ambiguity, for which whole genome sequencing was performed in the laboratory (Supplementary Table S1). We excluded three genomes from the NCBI database, which were apparently *C. jejuni* strains, falsely annotated as *C. coli* (GCA_001292485.1, GCA_001292205.1 and GCA_001292265.1). K-mers with direct matching were subtracted from the k-mer pool and the residual k-mers were compared against 152 complete *C. jejuni* genomes from the NCBI database and 3 additional sequences from the BfR strain collection (Supplementary Table S1). Those k-mers, which matched sequences present in at least 95% of the *C. jejuni* and maximal 5% of the *C. coli* genomes, were mapped against the reference *C. jejuni* strain NCTC 11168. Example k-mer mappings of a “hybrid” (>10% *C. jejuni* introgression) and a “half hybrid” strain (<10% *C. jejuni* introgression but with ambiguous qPCR result) against the NCTC 11168 reference sequence are depicted in Fig. 4. The recombination events of *C. jejuni* sequences in *C. coli* appear to be distributed all over the chromosome.

An analysis of the recombination size was performed with a minimal assumed recombination event size of 100 bp and various maximal gaps between events of 100–500 bp between k-mer matchings. As expected, the recombination size was increased with increasing size of maximal gaps. However, the overall median size of recombination events ranged between 297 and 512 bp and the maximal event was between 11.4 and 11.8 kb detected in strain BfR-CA-08318. This might hint at the potential of *C. coli* to incorporate large regions of more than 10 kb within one recombination event but that most of them were below 1 kb. The number of detected recombination events per strain ranged in median between 218 and 230 events. Note that our analysis might underestimate the number and the size due to the fact that only k-mers with exact and unique matches to the reference *C. jejuni* NCTC 11168 and to 95% of all *C. jejuni* strains included in the study were considered. A cumulative plot of recombination events (from gap size analysis of maximal 100 bp) in each strain sorted by the chromosomal location of the reference sequence is depicted in Fig. 4, indicating that common recombination events occurred in multiple *C. coli* hybrid strains and that the overall *C. jejuni* content in these strains was similar as analysed by k-mer analysis via the KmerFinder 3.1 of CGE.

### What has happened at *mapA* and *ceuE* loci

**Figure 5:**
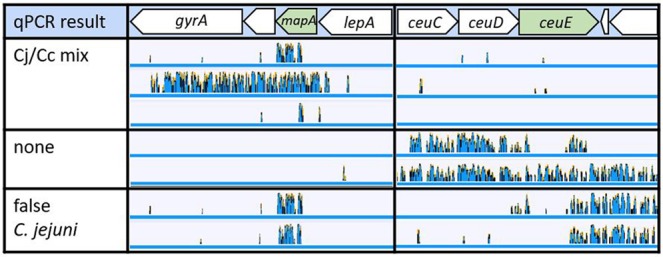
Strains with ambiguous qPCR result display various *C. jejuni* recombination events at the *mapA* and *ceuE* locus. In blue, coverage of k-mers to the reference sequence of *C. jejuni* NCTC 11168 of example strains with ambiguous qPCR results. Cj/Cc mix, integration of *C. jejuni* sequences at the *mapA* locus; none, integration of *C. jejuni* sequences in the *ceuE* locus; false *C. jejuni*, integration of *C. jejuni* sequences in both targets.

In order to find out, why qPCR results led to ambiguous and even false results, k-mer mapping of these isolates to *C. jejuni* NCTC 11168 was visualized with Geneious Prime. The k-mer analysis revealed different patterns of *C. jejuni* introgression in *C. coli mapA* and *ceuE* genes and their gene context (Fig. 5). In *mapA* either several small or large recombination events, covering also the adjacent 3′ genes Cj1028c and *gyrA* and the 5′ upstream located *lepA*, were identified. In the false positive *C. jejuni* isolates, the *ceuE* locus exhibited a mosaic allele structure with the 5′ start of *ceuE* displaying a typical *C. coli* sequence and the 3′ end matching *C. jejuni* sequences. In one strain with no amplification of any of both targets (“none”), the complete *ceuE*, including adjacent *ceuD* and *ceuC* and 3′ downstream located tRNA and Cj1356c were exchanged by *C. jejuni* sequences. Thus, although introgression of *C. jejuni* sequences into *C. coli mapA* locus was more frequently observed, the *ceuE* locus can also partially or fully be introgressed by *C. jejuni* DNA in contrast to previous observations^23^.

For a further analysis we looked at the annealing sites of the oligos used for *mapA* and *ceuE* amplification. A multiple alignment of the genes, displaying *C. jejuni* sequence content as identified by k-mer analysis, was done using MegAlign Pro 14 (Supplementary Fig. S1). As expected from the real-time PCR result, those strains from which both targets *mapA* and *ceuE* were amplified, exhibited a sequence at *mapA*, which is typical for *C. jejuni* but maintained a typical *C. coli ceuE* allele. Those four strains with no real-time PCR signal at all (“none” strains), had a *C. coli* allele at *mapA* but either a complete *C. jejuni* sequence of *ceuE* (BfR-CA-15489) or a mosaic gene as indicated in Fig. 5. Besides, this analysis corroborated the idea that independent recombination events led to similar outcome of the PCR result, since the sequence of the annealing sites of oligos and probes at the *mapA* locus was different in the strains with ambiguous PCR results and overall sequence of *mapA* and *ceuE* varied within PCR categories.

### Are the strains with ambiguous qPCR result typable by other PCR assays

There are various other PCR assays published for species differentiation of *C. jejuni* and *C. coli* with and without detection of further *Campylobacter* spp^12,24–29^. Thus, the whole-genome sequences of the strains leading to ambiguous species differentiation with *mapA*/*ceuE* targets^3,5^ and the correctly identified *C. coli* hybrids with high *C. jejuni* content were further assessed *in silico* (Supplementary Fig. S1). First, a second multiplex PCR targeting *mapA*/*ceuE* was evaluated^25^, leading to 56% ambiguous or false results. The sequence data revealed that also *cpn60* detection^24^ would lead to false species identification of all *C. coli* hybrid strains but would identify *C. coli* correctly in the “half hybrids”, in which *mapA*/*ceuE* were no reliable targets. Besides, strain BfR-CA-17110 harboured a *C. jejuni* sequence in the target *cadF*^28,29^, leading to false *C. jejuni* identification of this *C. coli* strain. All other targets *hipO*, *glyA*, *lpxA* and *ccoN* displayed either no or low *C. jejuni* introgression. If *C. jejuni* sequences were detected within the genes, the annealing sites of the PCR oligos were not affected, thus, a correct output of the PCR is expected.

As mentioned above, MLST typing based on the seven housekeeping genes *aspA*, *glyA*, *gltA*, *glnA*, *tkt*, *uncA*, *pgm* was impaired in the *C. coli* hybrid strains with high content of *C. jejuni* sequence introgression (Fig. 3 and Supplementary Table S1). In particular, *aspA* and *tkt* contained *C. jejuni* sequences in all hybrid strains for at least 38 or 26% gene coverage, respectively. *pgm* displayed *C. jejuni* sequences in 24 of the 29 hybrid strains between 13 and 29% of the gene length. Low amount of *C. jejuni* introgression up to 16% were found in *gltA*, *glnA* and *uncA* in 28 strains. *glyA* was the most “stable”, since all strains harboured a classical *C. coli* allele or displayed just one *C. jejuni* k-mer match, which was not significant.

### Which genes/locations were exchanged by *C. jejuni* sequences in the *C. coli* hybrid strains

**Figure 6:**
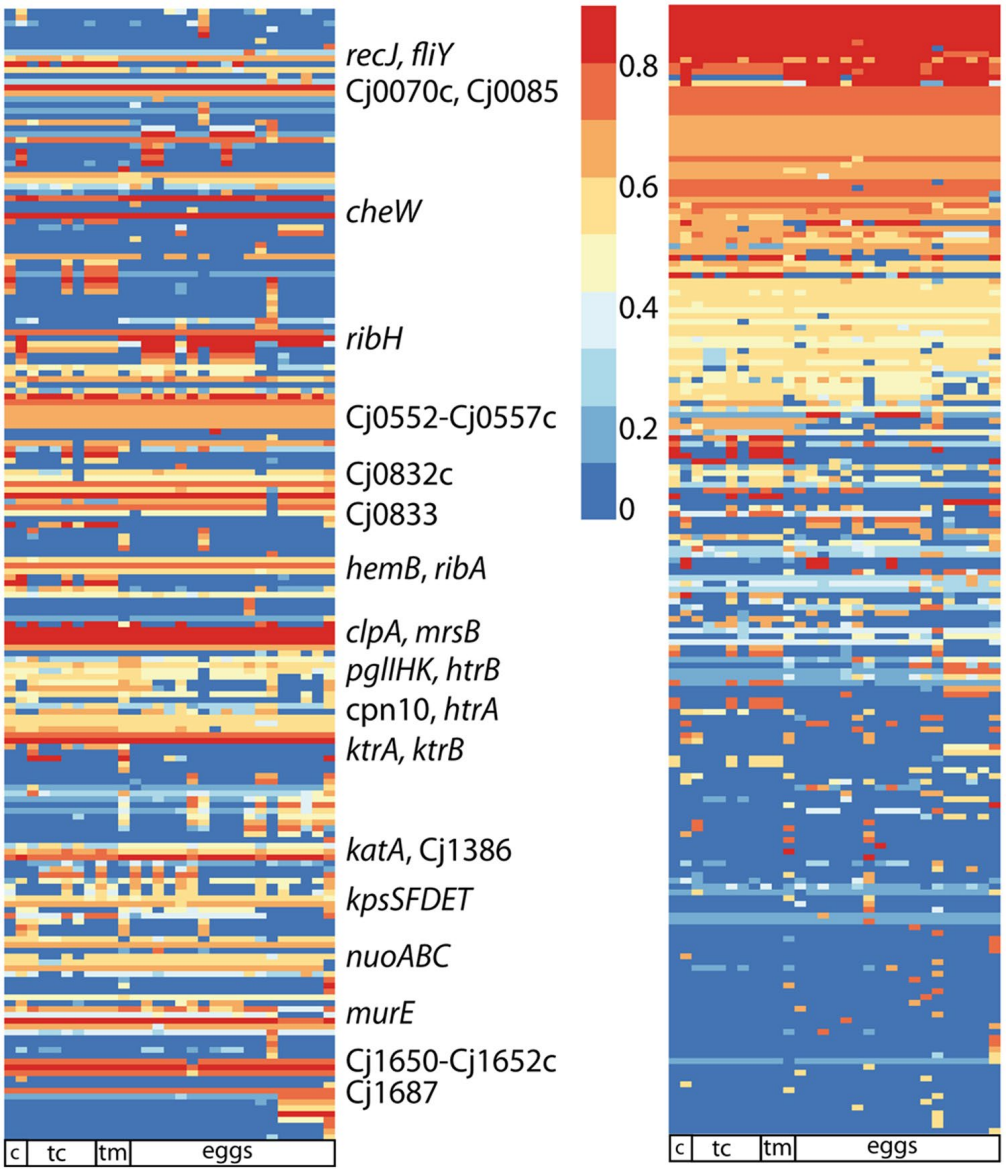
Cc/Cj hybrid strains share common genes with *C. jejuni* sequence content, which are distributed over the chromosome. Visualization of genes with at least 50% k-mer coverage of *C. jejuni* sequences identified in at least one of the 29 Cc/Cj hybrid strains (with >10% *C. jejuni* introgression). X-axis, strains; y-axis, genes. Left, heatmap sorted according to gene location in the reference *C. jejuni* sequence; right, heatmap sorted according to genes with high number of strains and high *C. jejuni* sequence exchange. Colours indicate coverage of gene length by *C. jejuni* sequence specific k-mer (16 bp) matches in % as detailed in the figure. Example genes with high coverage in the majority of strains are indicated. Below heatmaps, isolation source of the strains: c, chicken meat; tc, turkey cecum; tm, turkey meat and eggs.

Considering the typing results with some genes overrepresented for *C. jejuni* introgression (e. g. *cpn60* or *aspA*) and the observations of distinct recombination regions, the question arises, whether *C. jejuni* sequence exchange had common patterns in the 29 *C. coli* hybrid strains. 346 genes were covered by k-mers in at least 20% of the gene length in minimally one of the high content *C. coli* hybrid genomes. They were depicted as heatmap by the R package pheatmap v.1.0.12. (Supplementary Fig. S2). For better visualization (Fig. 6), the 194 genes were filtered to 50% of gene length exchange by *C. jejuni* sequences in minimally one of the hybrid strains. As shown above, recombination events, i. e. genes with *C. jejuni* content, were distributed throughout the chromosome. Intriguingly, similar genes were exchanged in multiple *C. coli* hybrid strains with similar extent of *C. jejuni* sequence exchange (same coloured lines in Fig. 6). Therefore, we looked at those genes, which were exchanged for at least 20% gene length coverage in at least 25 of the 29 strains and identified 104 genes, identified with both k-mer analysis (16 bp or 31 bp) and additional 8 and 14 genes with 16 bp and 31 bp k-mer analysis, respectively (Supplementary Table S2). Gene categories were annotated by EggNOG 4.5.1^30^ and, after integration of additional information on *Campylobacter* homologues, summarized in functional categories (Supplementary Table S2). Assuming that *C. jejuni* sequence introgression into *C. coli* strains was random, we simulated 300 recombination events in 800 core genes of 30 strains by using a Python script (available at https://gitlab.com/microbial_genomics/relative-kmer-project). As expected, and statistically proven significant (p < 0.05 by Chi^2^ test), the random distribution of genes recombined in multiple strains was distinct from the real distribution of observations in the hybrid strains (Supplementary Fig. S4). This supports our hypothesis of a selective process on the gene set affected by *C. jejuni* introgression in the hybrid strains.

Almost half of all identified genes encode proteins involved in oxidative stress response (*katA*, Cj1386, *mrsB*, *canB*, Cj0833c *hydA*, *hydA2*, *nadD*, *nuoA*, *nuoB*, *nuoC*, Cj0081), stress response in general (*clpA*, *htrB*, *htrA*, *cpn10*, *cpn60*), DNA metabolism and repair (*purF*, *pyrG*, *thyX*, *rarA*, *recJ*, *ung*, *ribA*, *guaB*, *dut*), chemotaxis and flagellar motor switch (*cheA*, *cheV*, *cheW*, *fliY*), signal transduction (Cj1110c, Cj1227c, Cj1258), membrane transporters (*crcB*, cj0832c, *ktrA*, *ktrB*, Cj1257c, Cj1687), cell wall and capsule biosynthesis (*kpsS*, *kpsE*, *kpsF*, *kpsD*, *kpsT*, *murE*) and *metK* encoding a S-adenosylmethionine transferase, involved in providing the substrate for methylation reactions. This suggests that *C. jejuni* sequence recombination in the *C. coli* hybrid genes was not random but might modulate the fitness of the *C. coli* hybrid strains, selected for survival in a harsh environment. Intriguingly, an American isolate, *C. coli* RM4661, from turkey carcass origin (NZ_CP007181.1) was identified as a Cc/Cj-hybrid strain, sharing 106 of the 126 *C. jejuni* introgressed genes revealed in the majority of our hybrid strains (Supplementary Table S2). We propose that this strain underwent a similar selection procedure, which corroborates our hypothesis of independent functional adaptation upon selection in a harsh environment.

### Which *C. jejuni* sequence exchange leads to amino acid exchange in the protein and might represent a functional adaptation in *C. coli*

We checked whether the gene variants of the *C. coli* hybrid strains lead to protein variants different from *C. coli* proteins. Since *C. jejuni* and *C. coli* proteins differ in average by nearly 40 amino acids^31^, it was expected that most of the observed *C. jejuni* introgression covering at least 20% of the gene length leads to changes in protein sequence. BLAST analysis (https://blast.ncbi.nlm.nih.gov) was performed on a subset of the above-mentioned identified gene translations and in all of the cases amino acid exchanges were detected in the hybrid variants as compared to *C. coli* typical protein sequence. It remains to be investigated in future studies, how these variations impact protein function with respect to *C. coli* survival capacity under stress conditions.

## Discussion

The German National Reference Laboratory for *Campylobacter* has access to a large collection of representative isolates from Germany. With this set of isolates in hand we were able to identify multiple strains with ambiguous species differentiation, in particular, isolated from eggs but also from poultry meat and turkey cecum. Further isolates from eggs showed that from this isolation source nearly half of all *C. coli* displayed an extended amount of *C. jejuni* sequences incorporated in the genome.

A study comparing *C. coli* clade 1 (ST-828 and ST-1150) from agriculture with nonagricultural unintrogressed *C. coli* clade 2 and 3 demonstrated the potential of incorporation of substantial *C. jejuni* sequences in clade 1 *C. coli*^31^. The authors identified 26 *C. jejuni* genes present in *C. coli* clade 1 but absent in clade 2 and 3. Our analysis focussed on *C. coli* hybrid strains as a fraction of clade 1 and deciphers ongoing extended *C. jejuni* introgression in these strains. As expected, we only have an overlap of 2 genes (Cj0555 and *htrB*) out of the identified 26 with the study of Sheppard *et al*.^31^, since we compared our hybrid strains against 113  *C. coli* sequences (mostly clade 1), including sequences from the NCBI database. This supports the notion that the *C. jejuni* recombination events found in this study represent a further development of *C. coli* strains. Since the *C. coli* hybrids were predominantly isolated from eggs, this supports the notion that the identified *C. jejuni* sequence incorporations might be a consequence of functional adaptation to survival in a harsh environment. *Campylobacter* is transmitted on egg shells via fecal contamination. On the shell, the bacterium encounters oxidative stress but also dryness and, thus, osmotic stress as well as nutrient and cold stress. Usually after 5–6 days, *Campylobacter* are no longer cultivatable from faeces^32,33^.

### Adaptation to harsh environment might explain shared *C. jejuni* recombinations in *C. coli* hybrids

The hybrid strains carried gene variants of *C. jejuni* or mosaic genes involved in oxidative stress response, such as katalase (*katA*) and Cj1386, which was shown to encode an atypical hemin-binding protein, mediating the trafficking of hemin to katalase^34^. Katalase is one of the key enzymes for protection against oxidative stress by cleaving peroxide to water and oxygen. *mrsB* (cj1112c) encoding a methionine sulphoxide reductase, was shown to protect *C. jejuni* against oxidative and nitrosative stress^35^. Furthermore, *canB* displayed *C. jejuni* sequences in the hybrid strains, encoding carbonic anhydrase, an enzyme important for growth at low CO_2_ concentrations^36^. A further oxidoreductase (Cj0833c) and genes encoding for the Ni/Fe hydrogenase small subunit *hydA* (Cj1267c) and *hydA*2 (Cj1399c) as well as *nadD* (Cj1404) involved in the synthesis of the redox cofactor NAD + were found to harbour *C. jejuni* sequences. Furthermore, *nuoA*, *nuoB*, *nuoC* implicated in transfer of electrons in the respiration chain and Cj0081, encoding the cyanide-resistant CioAB, which is proposed to lower oxygen levels and maintain microaerobic conditions^37^, were identified to bear *C. jejuni* sequences in the hybrid strains. The *htrB* gene encoding a lipid A acyltransferase was proposed to play a role in regulation of cell responses to environmental harsh conditions, such as acid, heat, oxidative and osmotic stress^38^. As mentioned above, also *htrA*, which encodes a protease and chaperone activity with roles in virulence and oxidative stress defence^39–41^ was among the genes with *C. jejuni* sequence detected in all hybrid strains. Besides *cgb*, encoding a single-domain haemoglobin, was suggested to protect *Campylobacter* against nitric oxide and nitrosative stress^42^.

Interestingly, also genes implicated in general stress response as the *clpA* ATPase and the chaperone genes *cpn10* and *cpn60* were affected by *C. jejuni* introgression. The latter *cpn60* (*groEL*) also serves as target for species differentiation^24^, inevitably leading to false species identification of the hybrid strains. Among the genes with *C. jejuni* introgression in the hybrid strains were several with roles in DNA metabolism and repair, such as *purF*, *pyrG*, *thyX*, *rarA*, *recJ*, *ung*, *ribA*, *guaB* and *dut*. Moreover, motility-associated genes, like the chemotaxis genes *cheA*, *cheV*, *cheW* and *fliY*, encoding a flagellar motor switch protein, displayed *C. jejuni* sequences.

In addition, our list of genes with *C. jejuni* content in the hybrid strains, also contained genes implicated in cell wall (*murE*) and capsule biosynthesis (*kpsS*, *kpsE*, *kpsF*, *kpsD*, *kpsT*). Consistently, in *C. jejuni* strains enhanced biofilm formation capacity, which might also be associated with enhanced survival under oxidative stress, was attributed to genes implicated in oxidative stress defence, motility, cell wall and capsular biosynthesis^43^.

We suggest that modification of genes by recombination of *C. jejuni* sequences in a common set of genes in most of the hybrid strains, might reflect selection of survivors from harsh environments.

### Practical implications for diagnostics

The hybrid strains can elude molecular typing, such as species differentiation using the *mapA*/*ceuE* targets and MLST. It was previously found that *mapA*/*ceuE* targets might lead to ambiguous qPCR results in six identified strains out of a data collection of around 1700 sequences^23^. In our study we identified in total 37 strains (21 “hybrid” strains and 16 “half hybrid” strains), which were not identifiable in the qPCR using *mapA*/*ceuE* targets, including two isolates, which were falsely identified as *C. jejuni*. All “hybrid” strains failed to be typed using the *cpn60* target^24^ and one “half-hybrid” would be incorrectly typed as *C. jejuni* using the *cadF* target^28,29^.

*C. jejuni* sequence introgression into *aspA* and adjacent regions (including Cj0081) was previously detected in two *C. coli* strains from turkey^44^. Our data showed that MLST as phylogenetic assay has limitations, since *C. jejuni* sequences were found in six of the seven housekeeping genes in the majority of “hybrid” strains. Hence, standardized typing methods should consider perturbations due to extended recombination activity in *Campylobacter*. Thus, it is recommendable to include multiple independent species differentiation methods as future molecular annex to ISO 10272-1/2:2017 and to be aware of phylogenetic bias in source attribution analysis.

### Conclusions and further aspects

There are various studies dealing with the differential survival of *C. jejuni* and *C. coli* under different environmental and host conditions. It has to be noted, that stress survival of the microaerobic *Campylobacter* is one of the major and still enigmatic topics in order to explain the pathogens widespread dissemination. *C. jejuni* was shown to survive longer in liver juice^45^. Aerotolerant *C. jejuni* strains were identified^46^ but also aerotolerant *C. coli* isolates were highly prevalent in other studies^47^. Survival in harsh environments might be a result of various factors and also dependent on the specific genomic background. In aerotolerant *C. coli* point mutations were detected in other genes, not obviously implicated in oxidative stress response^48^. Thus, it remains elusive, how *Campylobacter* species modulate their gene pool in order to adapt to changing environments. However, the identification of hybrid strains, mainly selected from a harsh environment, exhibiting an extended amount of *C. jejuni* sequences in a common gene set, shows the enormous potential of *Campylobacter* for extensive genetic exchange for fitness enhancement.

## Materials and Methods

### Strains and growth conditions

*C. jejuni* and *C. coli* field strains were isolated from different food matrices and animal samples by the Federal State Laboratories according to ISO 10272^11^. At the National Reference Laboratory, isolates were cultured on Columbia agar (Oxoid, Germany) supplemented with 5% sheep blood (Oxoid, Germany) (ColbA) or passaged through Bolton broth and subcultured on mCCDA in case strains still exhibited non-*Campylobacter* background flora. Incubation was performed for 48 h under microaerobic conditions (5% O_2_, 10% CO_2_, rest N_2_) at 42 °C. Strains were stored at −80 °C using the cryobank system (Mast Diagnostica GmbH, Germany). For DNA extraction strains from −80 °C stocks were grown on ColbA for 24 h under microaerobic conditions at 42 °C and once subcultured for another 24 ± 4 h prior to use.

### Species differentiation by PCR

DNA of the strains was extracted by resuspension of the cell pellet in 5% Chelex 100 resin (Bio-Rad Laboratories GmbH, Germany), followed by incubation for 15 min at 95 °C and subsequent centrifugation. The supernatant was used for PCR analysis. For detection and species identification a real-time PCR method, targeting either a *C. jejuni* specific fragment of the *mapA* gene, a *C. coli* specific fragment of the *ceuE* gene or a *C. lari* specific fragment of the *glyA* gene was performed^4,5^. In case of ambiguity of the results, a second gel-based multiplex-PCR was applied, targeting specific fragments of the *hipO* gene for *C. jejuni*, the *glyA* gene for *C. coli* and *C. upsaliensis*, the *cpn60* for *C. lari*, the *sapB2* gene of *C. fetus* and a Campylobacterales specific fragment of the 23 S rRNA gene^12^.

### Matrix-assisted laser desorption/ionization (MALDI-TOF) analysis

Colony material of a 24 h ColbA plate was spotted onto the target plate (MSP 96 target polished steel (MicroScout Target) plate; Bruker Daltonik, Germany). After air drying the spots were overlaid with 1 µl of saturated α-cyano-4-hydroxy-cinnamic acid matrix solution (200 mg in 2.5% trifluoroacetic acid/50% acetonitrile) and dried completely. MALDI-TOF MS analysis was performed using MALDI-TOF Microflex LT (Bruker Daltonics, Germany) using a range of 2,000–20,000 m/z (mass to charge ratio) following the calibration with Bacterial Test Standard (Bruker Daltonics, Germany). For each spectrum 240 laser shots were summed up in 40 shot steps, but at least 80 shots per raster spot from different positions within the sample were acquired by the AutoXecute method using the software FlexAnalysis 3.4. The spectra were compared with the MBT Compass Library, Revision F (Bruker Daltonics, Germany). Each identification obtains a score value. The identification at the species level with a score ≥ 2.000 was considered correct^16–18^.

### Whole genome sequence analysis

*Campylobacter* strains grown on ColbA for 24 h under microaerobic atmosphere at 42 °C were harvested and DNA was extracted using the PureLink Genomic DNA Mini Kit (Thermo Fisher Scientific, USA). The quality of the DNA was evaluated by spectral analysis (NanoDrop Spectrophotometer, Thermo Fisher Scientific, USA) and the concentration was fluorimetrically quantified by Qubit 3.0 Fluorometer (dsDNA HS Assay Kit 0.2–100 ng; Thermo Fisher Scientific, USA). DNA libraries were prepared using the Nextera XT DNA Library Prep kit or the Nextera DNA Flex Library Prep Kit according to the manufacturer’s instructions (Illumina, San Diego, USA). Quality of the libraries was assessed by gel analysis or on a fragment analyser 3408 (Advanced Analytical Technologies Inc., USA). Paired-end sequencing was performed on the Illumina MiSeq (2×301 cycles) or the NextSeq (2×151 cycles) platform using the MiSeq v3 (600 cycles) reagent kit or the NextSeq 500/550 Mid Output kit v2.5 (300 cycles), respectively. The sequences were published within the BioProject No. PRJNA595957, BioSample No. SAMN13577876-SAMN13577920, SRA accession No. SRR10698060-SRR10698104 at NCBI sequence read archive (SRA). New MLST alleles and MLST-ST types were uploaded to PubMLST.

### Sequence analysis

Sequences were analyzed by Ridom Seqsphere+ v. 6.0.0 (2019–04) (Ridom, Muenster, Germany) using the cgMLST scheme of 1343 gene targets previously proposed^22^, with 98% required identity and 98% required percentage of coverage to one of the known alleles (allele library status June 2018). Quality trimming was performed in a window of 20 bp with Phred score 30. The obtained average coverage (processed, unassembled) was >75-fold. Raw reads were *de-novo* assembled via SPAdes 3.11.1^49^ with careful option, which performs a mismatch correction. The number of assembled contigs was between 31 and 130, the total size of the assemblies ranged from 1.65 to 1.92 Mb. At least 95% “good targets” were found for cgMLST-based analysis using the previously proposed cgMLST scheme^22^. Average nucleotide identity (ANI) analysis was done using the tool FastANI^19^. Core genome phylogeny was calculated using Roary v.3.12.0^50^ with a sequence identity of at least 80%. This resulted in 800 core genes that were used to build a phylogenetic tree with RAxML v.8.2.10^51^ (100 bootstraps). Finally, the phylogenetic tree was adjusted for recombination sites using ClonalFrameML v.1.11^52^.

For prescreening of sequences for *C. jejuni* introgression, assembled contigs were analyzed on the web-based KmerFinder 3.1 (Center for Genomic Epidemiology, DTU, Denmark)^13–15^, which splits the assembly contigs into overlapping 16-mers and searches for homology matches in sequenced bacterial organisms, filtered on coding sequences (CDS; starting with ATG). The percentage of k-mers matching to distinct reference genomes was received as output data. An in-depth k-mer analysis was performed using an in-house pipeline. For this purpose the assemblies from Ridom Seqsphere+ after SPAdes assembly were used. A k-mer based databases from assembled and closed genomes from *C. jejuni* and *C. coli* (Supplementary Table S1) were built by kmc v.3^53^ with a k-mer size of 16 bases or 31 bases. In order to identify *C. jejuni* specific genes, the database of *C. jejuni* includes k-mers present in at least 95% of the genomes whereas the *C. coli* database contains k-mers present in at least 5% of the genomes. K-mers from mixed isolates were subsequently counted and compared against the databases with kmc_tools. K-mers present in *C. jejuni* database and absent in *C. coli* genomes were mapped against a closed reference strain NCTC 11168 (NC_002163.1) of *C. jejuni* by bowtie2^54^ and corresponding genes were identified with BEDTools v2.27.1^55^. Since read mappers are optimized for longer sequences, k-mers of size 16 bp were only considered for exact and unique matches for further downstream analysis. Since k-mers of size 31 bp are more specific, mapping results were not filtered at this step.

Genes covered by k-mers in at least 20% or 50% of the gene length and in one of the high content *C. coli* hybrid genome were visualized as heatmap by the R package pheatmap v.1.0.12. K-mers matched in *C. coli* hybrid genomes (as.bam files) were visualized in Geneious v.2019.2.1 using *C. jejuni* strain NCTC 11168 (NC_002163.1) as reference. Source code and scripts used to perform those steps are freely available at https://gitlab.com/microbial_genomics/relative-kmer-project.

## Supplementary information

Supplementary Figures.

Supplementary Table S1.

Supplementary Table S2.
