# Supplementary material for: Whole genome sequencing reveals extended natural transformation in Campylobacter impacting diagnostics and the pathogens adaptive potential
Source: Sci Rep. 2020 Feb 28;10:3686. doi: 10.1038/s41598-020-60320-y (PMC7048796; doi:10.1038/s41598-020-60320-y)
Supplement: Supplementary file 1 — Supplementary Figures. [file 41598_2020_60320_MOESM1_ESM.pdf]

Supplementary Figures

Whole genome sequencing reveals extended natural transformation in *Campylobacter* impacting diagnostics and the pathogens adaptive potential

Running title: WGS analysis of *Campylobacter* hybrid strains

Julia C. Golz, Lennard Epping, Marie-Theres Knüver, Maria Borowiak, Felix Hartkopf,

Carlus Deneke, Burkhard Malorny, Torsten Semmler, Kerstin Stingl

Suppl. Fig. S1. Alignment of target sequences from hybrid and half hybrid strains used for species differentiation in various published PCR assays. Primers and probes are aligned to target sequences of reference strains *C. jejuni* NCTC 11168 and *C. coli* BfR-CA-09557. Hybrids and half-hybrids with an introgression of ≥ 1 k-mer in the target gene were analyzed. Identified variants in the respective strains are detailed in Suppl. Tab. S1. Ruler 1, base location of target sequences of NCTC 11168 and in g) of BfR-CA-09557. a) *mapA* target according to [3,5]; b) *ceuE* target according to [3,5]; c) *mapA* target according to [25]; d) *ceuE* target according to [25]; e) *cpn60* target according to [24]; f) *cadF* target according to [29]; g) *cadF* target according to [28].

Suppl. Fig. S1

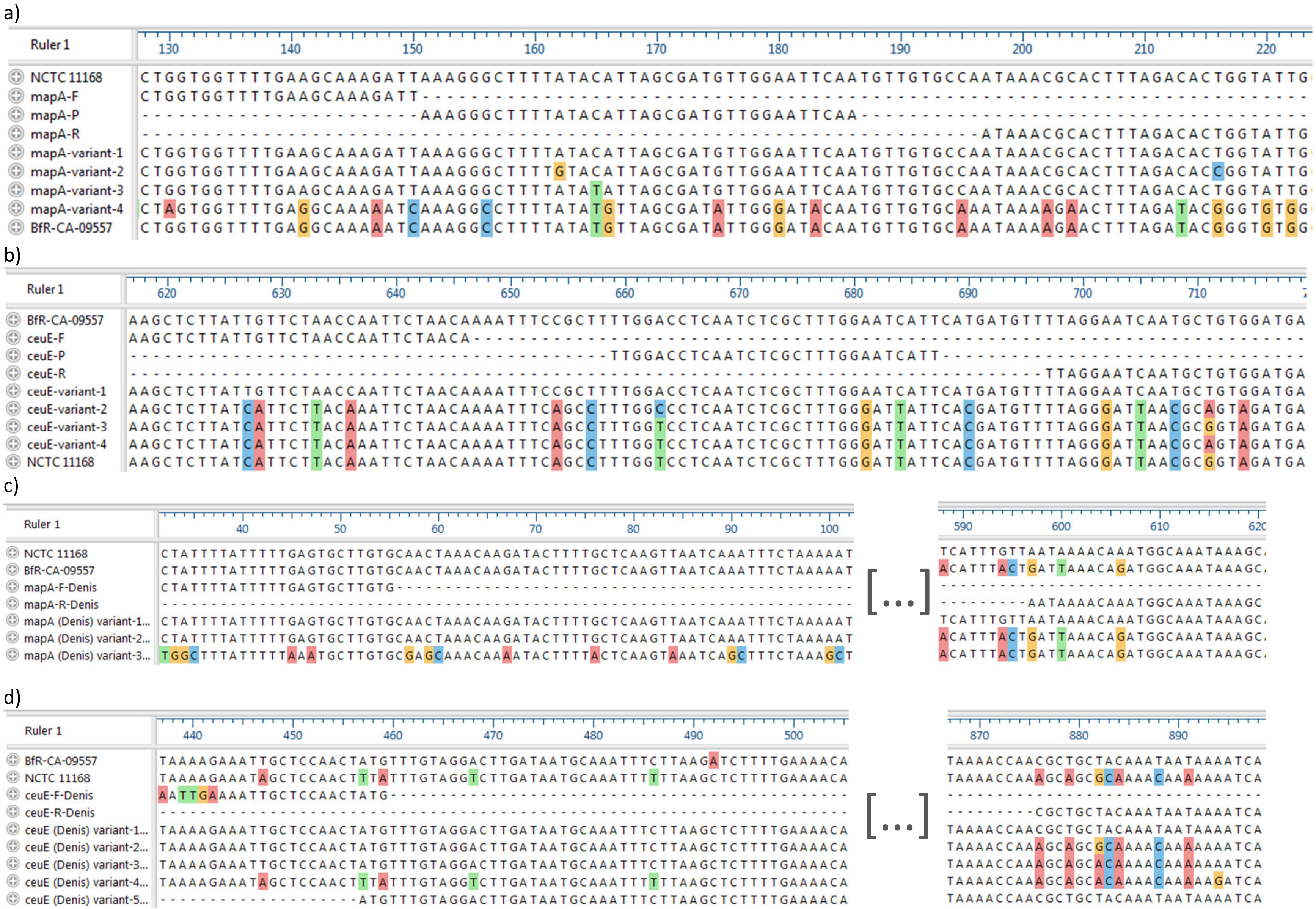
a)
b)
c)
d)
Ruler 1
NCTC 11168
mapA-F
mapA-P
mapA-R
mapA-variant-1
mapA-variant-2
mapA-variant-3
mapA-variant-4
BfR-CA-09557
ceuE-F
ceuE-P
ceuE-R
ceuE-variant-1
ceuE-variant-2
ceuE-variant-3
ceuE-variant-4
mapA-F-Denis
mapA-R-Denis
ceuE-F-Denis
ceuE-R-Denis
[…]

e)

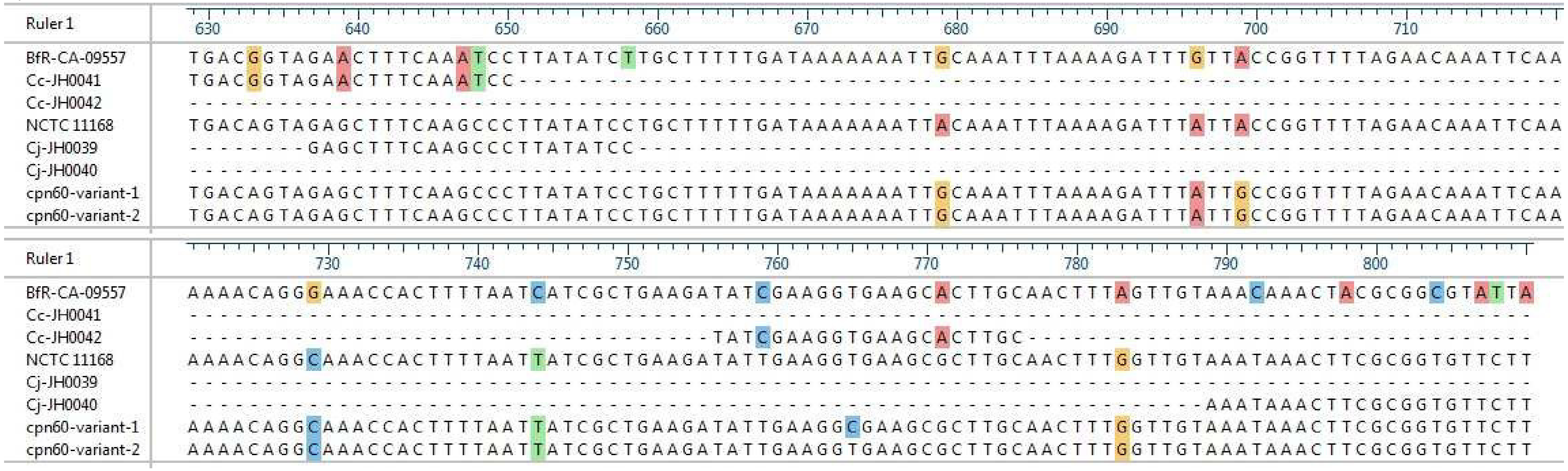
Ruler 1
630
640
650
660
670
680
690
700
710
BfR-CA-09557
Cc-JH0041
Cc-JH0042
NCTC 11168
Cj-JH0039
Cj-JH0040
cpn60-variant-1
cpn60-variant-2
Ruler 1
730
740
750
760
770
780
790
800
BfR-CA-09557
Cc-JH0041
Cc-JH0042
NCTC 11168
Cj-JH0039
Cj-JH0040
cpn60-variant-1
cpn60-variant-2

f)

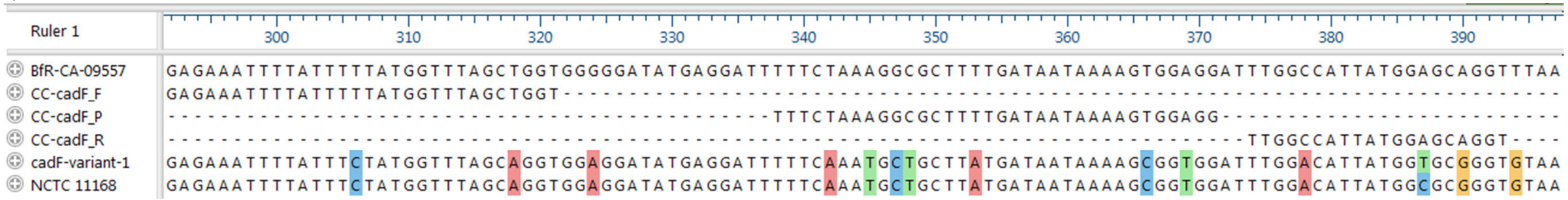
Ruler 1
300
310
320
330
340
350
360
370
380
390
BfR-CA-09557
CC-cadF_F
CC-cadF_P
CC-cadF_R
cadF-variant-1
NCTC 11168

g)

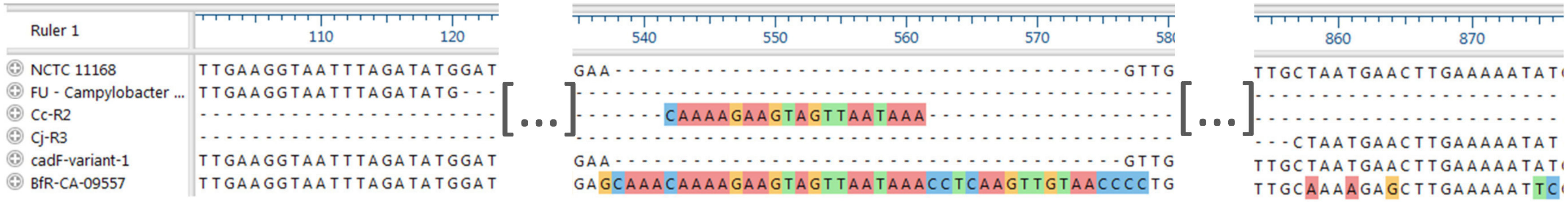
Ruler 1
110
120
540
550
560
570
860
870
NCTC 11168
FU - Campylobacter ...
Cc-R2
Cj-R3
cadF-variant-1
BfR-CA-09557
[...]
[...]

Suppl. Fig. S2. Heatmap visualization of genes with at least 20 % k-mer coverage of *C. jejuni* sequences identified in at least one of the 29 Cc/Cj hybrid strains (with >10 % *C. jejuni* introgression) X-axis, strains; y-axis, genes, sorted according to gene location in the reference *C. jejuni* NCTC 11168 sequence. Colours indicate coverage of gene length by *C. jejuni* sequence specific k-mers (16 bp) in % as detailed in the figure.

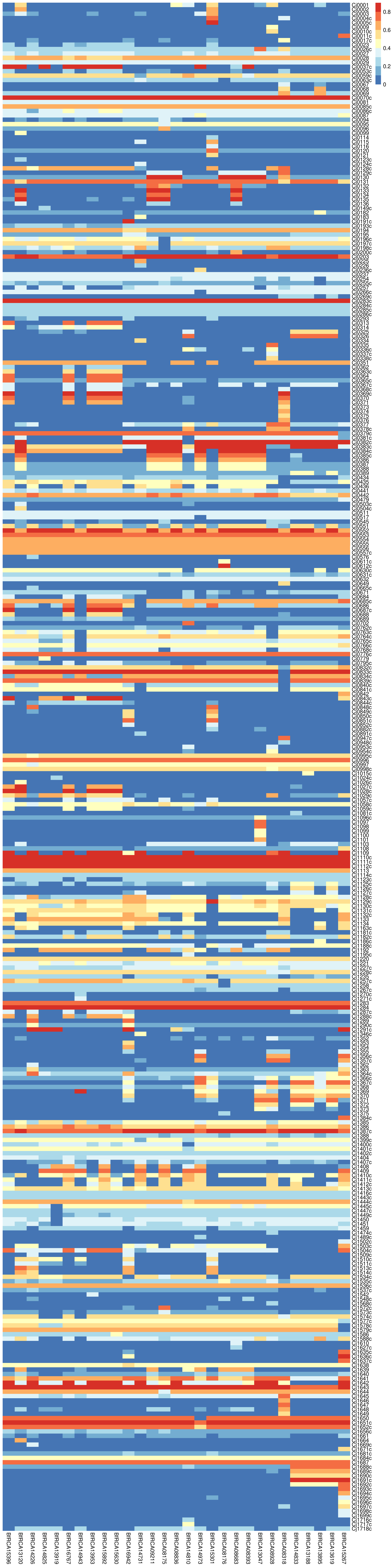

0.8
0.6
0.4
0.2
0
BfRCA15396
BfRCA13120
BfRCA14226
BfRCA14825
BfRCA13919
BfRCA16767
BfRCA14943
BfRCA13953
BfRCA15892
BfRCA15630
BfRCA16942
BfRCA14731
BfRCA09211
BfRCA08175
BfRCA08836
BfRCA14810
BfRCA14973
BfRCA15301
BfRCA08176
BfRCA08683
BfRCA08393
BfRCA13047
BfRCA08928
BfRCA08318
BfRCA14833
BfRCA13188
BfRCA13895
BfRCA13619
BfRCA15267

Suppl. Fig. S3. Heatmap visualization of genes with at least 20 % k-mer coverage of *C. jejuni* sequences identified in at least one of the 16 half hybrid strains (with <10 % *C. jejuni* introgression but with ambigious qPCR result). X-axis, strains; y-axis, genes, sorted according to gene location in the reference *C. jejuni* NCTC 11168 sequence. Colours indicate coverage of gene length by *C. jejuni* sequence specific k-mers (16 bp) in % as detailed in the figure.

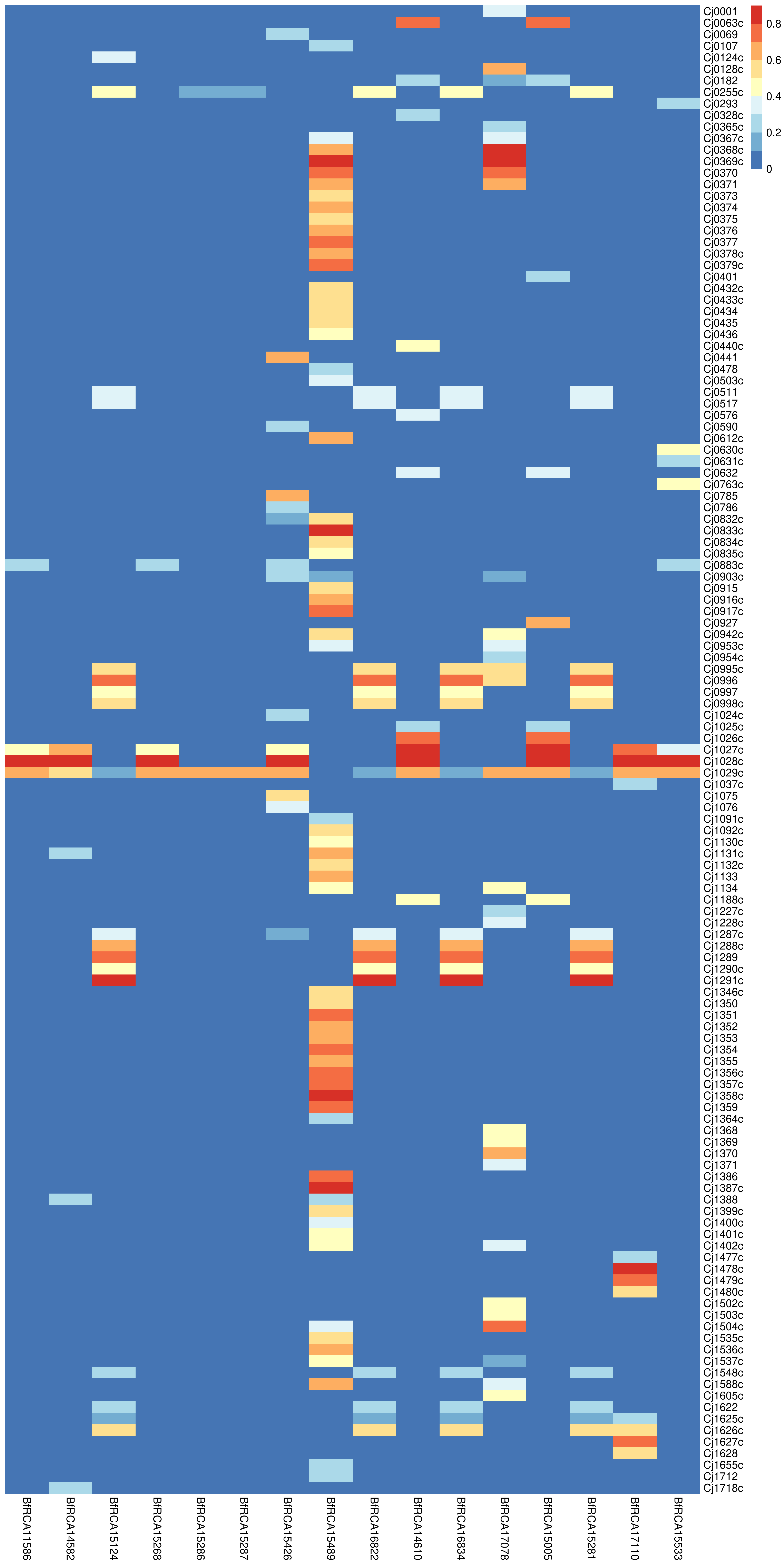
Cj0001
Cj0063c
Cj0069
Cj0107
Cj0124c
Cj0128c
Cj0182
Cj0255c
Cj0293
Cj0328c
Cj0365c
Cj0367c
Cj0368c
Cj0369c
Cj0370
Cj0371
Cj0373
Cj0374
Cj0375
Cj0376
Cj0377
Cj0378c
Cj0379c
Cj0401
Cj0432c
Cj0433c
Cj0434
Cj0435
Cj0436
Cj0440c
Cj0441
Cj0478
Cj0503c
Cj0511
Cj0517
Cj0576
Cj0590
Cj0612c
Cj0630c
Cj0631c
Cj0632
Cj0763c
Cj0785
Cj0786
Cj0832c
Cj0833c
Cj0834c
Cj0835c
Cj0883c
Cj0903c
Cj0915
Cj0916c
Cj0917c
Cj0927
Cj0942c
Cj0953c
Cj0954c
Cj0995c
Cj0996
Cj0997
Cj0998c
Cj1024c
Cj1025c
Cj1026c
Cj1027c
Cj1028c
Cj1029c
Cj1037c
Cj1075
Cj1076
Cj1091c
Cj1092c
Cj1130c
Cj1131c
Cj1132c
Cj1133
Cj1134
Cj1188c
Cj1227c
Cj1228c
Cj1287c
Cj1288c
Cj1289
Cj1290c
Cj1291c
Cj1346c
Cj1350
Cj1351
Cj1352
Cj1353
Cj1354
Cj1355
Cj1356c
Cj1357c
Cj1358c
Cj1359
Cj1364c
Cj1368
Cj1369
Cj1370
Cj1371
Cj1386
Cj1387c
Cj1388
Cj1399c
Cj1400c
Cj1401c
Cj1402c
Cj1477c
Cj1478c
Cj1479c
Cj1480c
Cj1502c
Cj1503c
Cj1504c
Cj1535c
Cj1536c
Cj1537c
Cj1548c
Cj1588c
Cj1605c
Cj1622
Cj1625c
Cj1626c
Cj1627c
Cj1628
Cj1655c
Cj1712
Cj1718c
BfRCA11586
BfRCA14582
BfRCA15124
BfRCA15268
BfRCA15286
BfRCA15287
BfRCA15426
BfRCA15489
BfRCA16822
BfRCA14610
BfRCA16834
BfRCA17078
BfRCA15005
BfRCA15281
BfRCA17110
BfRCA15533
0.8
0.6
0.4
0.2
0

Suppl. Fig. S4. The distribution of simulated random recombination events in *C. coli* core genes (green) is significantly different from that of the observed recombination events in the hybrid strains (purple). This is indicative of a selection process (i. e. functional adaptation) after recombination.

Suppl. Fig. S4

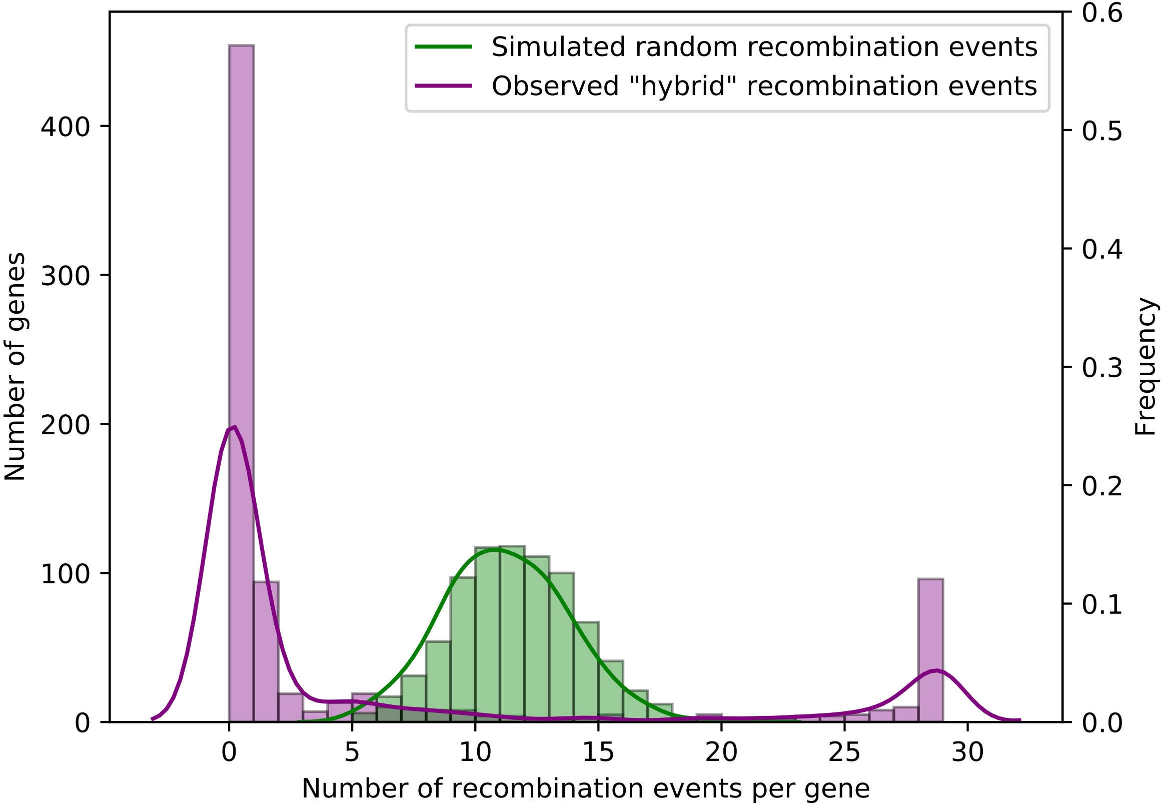

Simulated random recombination events
Observed "hybrid" recombination events
Number of genes
Frequency
Number of recombination events per gene
0
100
200
300
400
0.0
0.1
0.2
0.3
0.4
0.5
0.6
0
5
10
15
20
25
30
